# Supplementary material for: Interferon Gamma-Inducible NAMPT in Melanoma Cells Serves as a Mechanism of Resistance to Enhance Tumor Growth
Source: Cancers (Basel). 2023 Feb 23;15(5):1411. doi: 10.3390/cancers15051411 (PMC10000695; doi:10.3390/cancers15051411)
Supplement: Supplementary file 1 [file cancers-15-01411-s001.zip › cancers-2147716-supplementary.pdf]

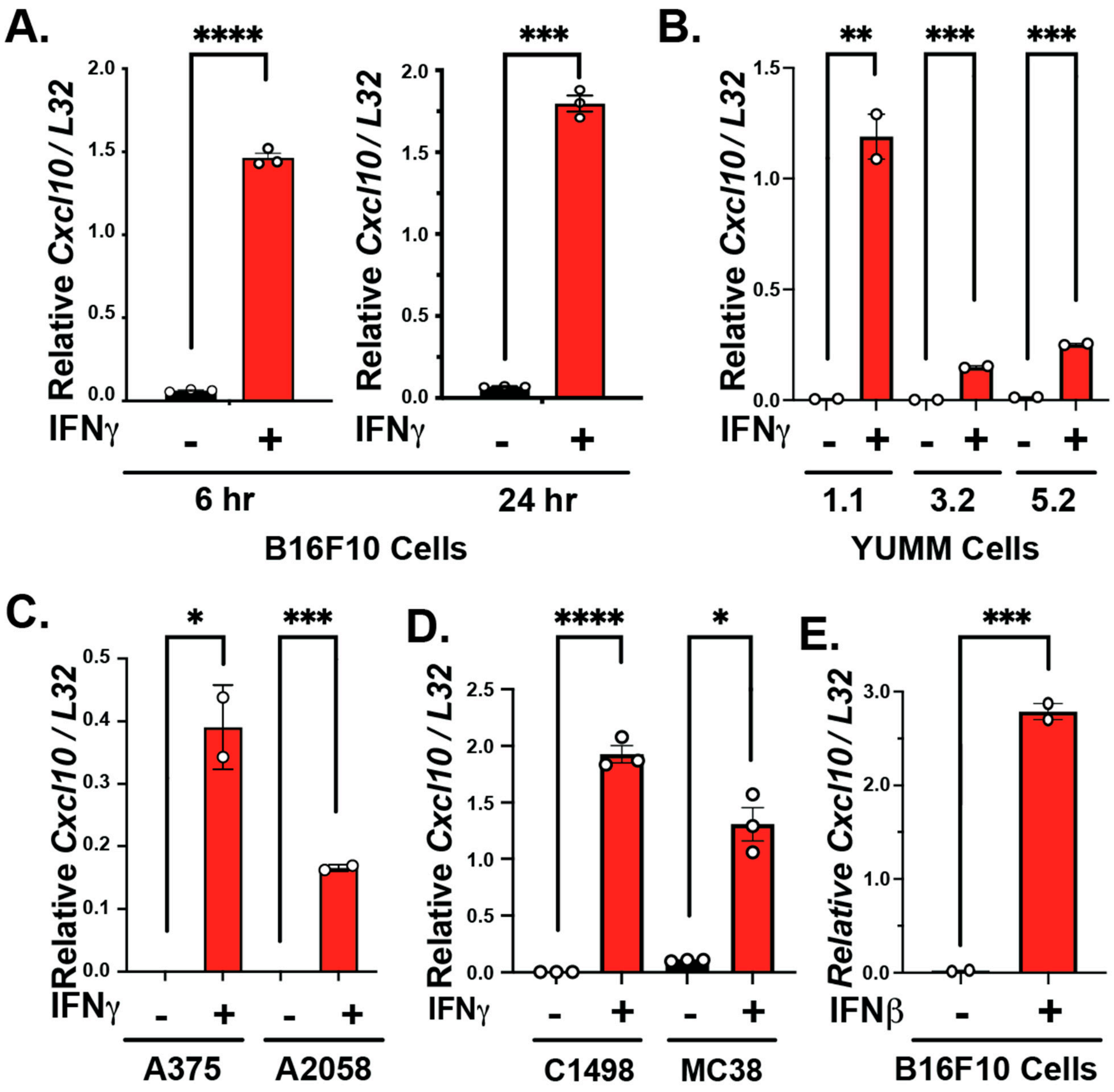

Supplemental Figure S1: IFNs induce CXCL10 in response to IFNγ in melanoma cells. B16F10 treated with IFNγ for 6 hours and 24 hours induce Cxcl10 RNA gene expression (A). Yale University Mouse Melanoma (YUMM) (B) and human melanoma cells (C) all induce CXCL10 RNA gene expression when treated with IFNγ for 6 hours. C1498 (leukemia) and MC38 (colon) cancer cells were treated with 6 hours of IFNγ and Cxcl10 RNA gene expression was measured (D). Type I IFN, IFNβ, leads to induced Cxcl10 RNA gene expression in B16F10 cells (E). Representative data from two experiments, n=2-3, unpaired t test, center values are mean ± SEM *$p \leq .05$, **$p \leq 0.01$, ***$p \leq 0.001$, ****$p \leq 0.0001$.

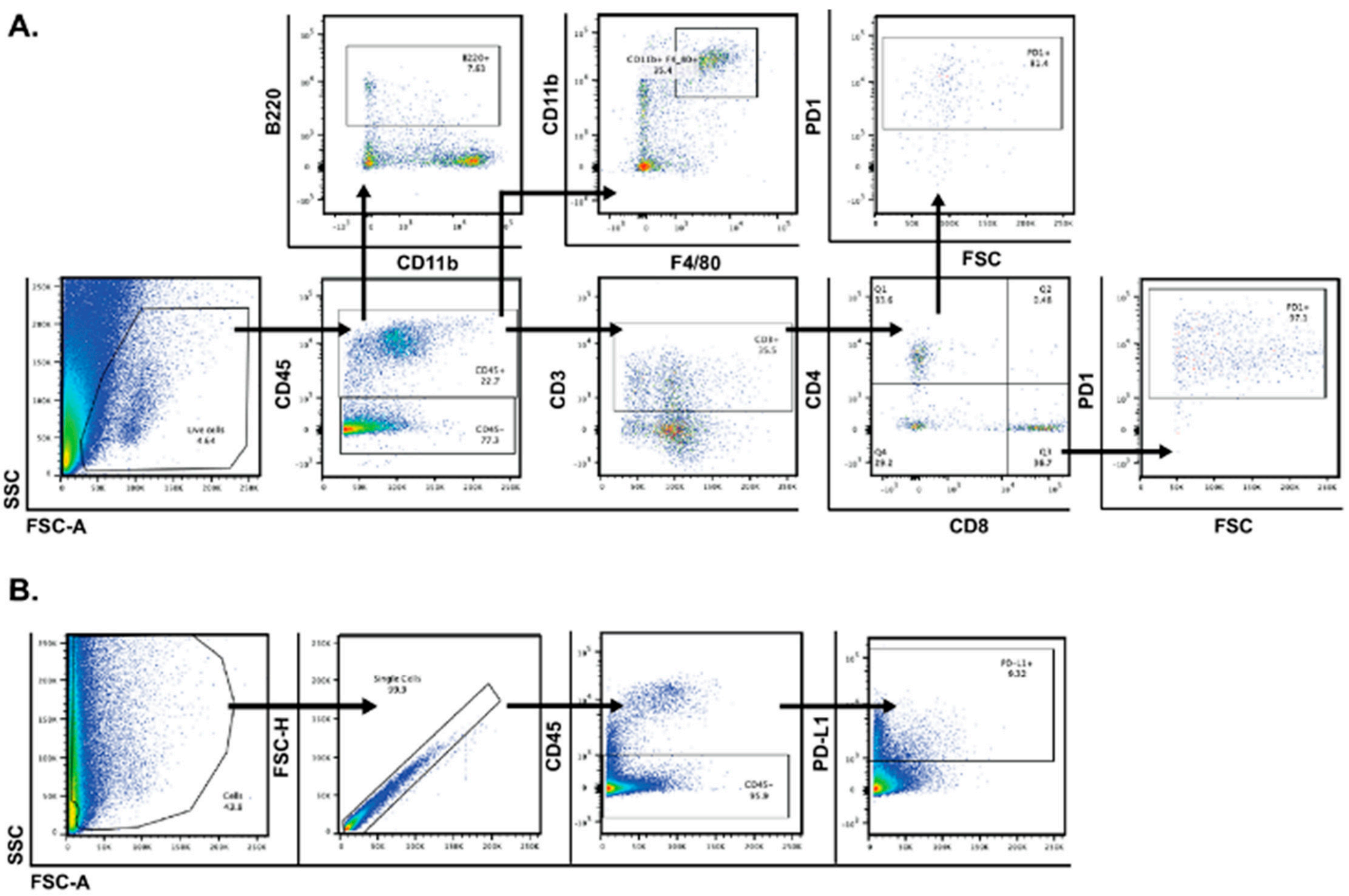

Supplemental Figure S2: Gating strategy for tumor and immune cells. B16F10 tumors were injected into mice and harvested for flow cytometry. Gating strategy to characterize immune (A) and non-immune (B) cells is shown above.

Table S1. Primer List

	Gene Name	Direction	Organism	Primer Sequence
qPCR	Nampt	F	Mouse	TACAGAGGCACCACTAATCATCAGAC
	Nampt	R	Mouse	CACGCCATCTCCTTGAATGACTCTA
	CXCL10	F	Mouse	CCAAGTGCTGCCGTCATTTTC
	CXCL10	R	Mouse	GGCTCGCAGGGATGATTTCAA
	RpL32	F	Mouse	ATCAGGCACCAGTCAGACC
	RpL32	R	Mouse	TTGAACCTTCTCCGCACCC
	Nampt	F	Human	taaaagctgttcctgagggct
	Nampt	R	Human	agaatttgtggccactgtgattg
	CXCL10	F	Human	GTGGCATTCAAGGAGTACCTC
	CXCL10	R	Human	TGATGGCCTTCGATTCTGGATT
	RpL32	F	Human	ATAGACGCACCATTGAAGTTAACCG
	RpL32	R	Human	CACCTTTTCATAGCAGTAGGCACA

Illumina Seq	Nampt	F	Mouse	ACACTCTTTCCCTACACGACGCTCTTCCGATCTGCGTCTGATTCAGCTGTCCCACTC
	Nampt	R	Mouse	GACTGGAGTTCAGACGTGTGCTCTTCCGATCTGGCGGGCATTTCTTCATCATTCCTC
sgRNA	Control gRNA		Mouse	GCTTTCACGGAGGTTCGACG
	Control gRNA		Mouse	ATGTTGCAGTTCGGCTCGAT
	SBS KO gRNA		Mouse	gACTACGTGATTTCTGTGAAA
	SBS KO gRNA		Mouse	gATAACCCTGGAGAATTTGTG

Table S2. Flow Cytometry Antibody List

Target	Fluorophore	Company	Catalog #
Annexin V	Pacific Blue	Biolegend	640918
B220	BV510	Biolegend	103247
Cell Trace Violet	Pacific Blue	Fisher Scientific	NC0402709
CD45	Pacific Blue	Biolegend	103126
CD3	APC/Cy 7	Biolegend	100222
CD4	FITC	Biolegend	100406
CD8	PE/Dazzle 594	Biolegend	100761
CD11b	APC	Tonbo Biosciences	60-0112-U100
F4/80	PE	Biolegend	123114
PD1	PeCy7	Biolegend	109110
PDL1	BV605	Biolegend	124321
7AAD	PerCP Cy5.5	Biolegend	420404
